# Yemeni refugees’ health literacy and experience with the Dutch healthcare system: a qualitative study

**DOI:** 10.1186/s12889-023-15732-6

**Published:** 2023-05-18

**Authors:** Abdulhakeem Al-Tamimi, Martina Parić, Wim Groot, Milena Pavlova

**Affiliations:** 1 Department of International Health, Care and Public Health Research Institute CAPHRI, Faculty of Health, Medicine and Life Sciences, Maastricht University, Maastricht, 6229 GT The Netherlands; 2 Department of Health Services Research, Care and Public Health Research Institute CAPHRI, Faculty of Health, Medicine and Life Sciences, Maastricht University, Maastricht, 6229 GT Netherlands; 3 School of Business and Economics, Maastricht University, Maastricht, 6211 LM Netherlands; 4 Maastricht Graduate School of Governance. Maastricht University, Maastricht, 6211 AX Netherlands

**Keywords:** Access to healthcare, Cultural competence, Health literacy, Health systems, Migrant health, Refugees, Yemeni refugees

## Abstract

**Background:**

The Netherlands is receiving increasing numbers of Yemeni refugees due to the ongoing war in Yemen. Since there is a lack of knowledge about access to healthcare by refugees, this study investigates the experiences of Yemeni refugees with the Dutch healthcare system from a health literacy perspective.

**Methods:**

Qualitative semi-structured in-depth interviews were conducted among 13 Yemeni refugees in the Netherlands, to gauge their level of health literacy and investigate their experiences with the Dutch healthcare system. Participants were invited using convenience and snowball sampling. Interviews were done in Arabic and then transcribed and translated ad verbatim to English. Deductive thematic analysis was conducted on the transcribed interviews based on the Health Literacy framework.

**Results:**

The participants knew how to use primary and emergency care, and were aware of health problems related to smoking, physical inactivity, and an unhealthy diet. However, some participants lacked an understanding of health insurance schemes, vaccination, and food labels. They also experienced language barriers during the first months after arrival. Furthermore, participants preferred to postpone seeking mental healthcare. They also showed mistrust towards general practitioners and perceived them as uncaring and hard to convince of their health complaints.

**Conclusion:**

Yemeni refugees in our study are well-acquainted with many aspects of Dutch healthcare, disease prevention, and health promotion. However, trust in healthcare providers, vaccination literacy and mental health awareness must improve, as also confirmed by other studies. Therefore, it is suggested to ensure appropriate cultural mediation services available for refugees as well as training for healthcare providers focused on understanding cultural diversity, developing cultural competence and intercultural communication. This is crucial to prevent health inequalities, improve trust in the healthcare system and tackle unmet health needs regarding mental healthcare, access to primary care, and vaccination.

**Supplementary Information:**

The online version contains supplementary material available at 10.1186/s12889-023-15732-6.

## Background

Healthcare systems have become increasingly complex due to demographic changes, technological developments, and globalization [1]. This makes it difficult for patients to navigate these systems. Therefore, a good level of health literacy (HL) is necessary. HL can be defined as [2]*“people’s knowledge, motivation and competences to access, understand, appraise, and apply health information in order to make judgments and take decisions in everyday life concerning healthcare, disease prevention and health promotion to maintain or improve quality of life during the life course*”[2]. There is plenty of evidence regarding the relationship between high HL on the one hand, and individual and social healthcare benefits on the other hand [2–4]. To illustrate, HL is associated with lower healthcare costs, better health outcomes, a healthier lifestyle, and improved self-efficacy [5].

[2]In the last decade, the European Union (EU) has been labelled as the “*global migration magnet*” [6]. Between 2015 and 2016, over 1.2 million asylum seekers crossed the EU borders, which is an unprecedented number since World War II [7]. Recently, the Dutch government was informed that almost half of the Yemeni refugees in Europe prefer the Netherlands due to high asylum acceptance rates and good living conditions [8]. This trend may continue as Yemen is hit by the ongoing conflict entering its ninth year, contributing to an economic crisis in 2023 [9]. More than 21 million Yemenis depend on humanitarian aid, with 17 million facing death, disease, hunger, and severe food insecurity [9].

This study focuses on the HL of Yemeni refugees in the Netherlands. This is an important refugee group to focus on because the Dutch government reported an increasing number of Yemeni asylum seekers, with around 2000 applications between April 2021 and April 2022 [10]. Refugees are asylum seekers whose applications meet the requirements for protection and are entitled to long-term residence and future naturalisation [11]. While the term “migrants” is occasionally used in this paper when a broader definition is needed, the refugee group is the main focus of our investigation, which represents a unique subset of migrants with special healthcare needs [11–13].

Generally, native populations have higher HL levels compared to their non-native counterparts [12]. This HL disparity contributes to health inequality between the two population groups [12, 13]. Furthermore, low HL is significantly associated with more healthcare utilisation and costs, such as longer hospitalisation, more general practitioner (GP) consultations, and higher emergency care costs [14]. One systematic review concluded that inadequate HL results in lower patient empowerment, leading to misuse of healthcare resources when caregivers are overdependent on medicalisation and biomedical approaches [15]. For example, 65% of refugees in Sweden have limited HL and 36% of them refrain from utilising healthcare due to language barriers, perception of helplessness about the benefit, and the preference to wait [13]. In fact, considerable evidence demonstrates that poor HL can obstruct access to healthcare by preventing the individual from seeking appropriate care and reducing treatment compliance and self-care [15].

Previous studies in the Netherlands show that settled ethnic minorities, e.g., those from Morocco, Turkey, Surinam, and the Netherlands Antilles, use GP services more frequently. They have 33% higher GP visits and a lower HL compared to the native Dutch population [16]. Moreover, these studies about HL among migrants in the Netherlands recommend that more research is necessary to explore how HL inequality between migrants and non-migrants develops [12, 16]. While there is data available on older ethnic minorities, there is a lack of knowledge on HL among newly arrived refugees [13].

Migration flows necessitate that health professionals pay attention to cultural sensitivities and the special needs of migrant populations to ensure quality and responsiveness in healthcare. This can be done by obtaining more evidence on HL among migrants because a ‘one-size-fits-all’ approach is oftentimes ineffective [17]. Therefore, this study investigates HL among Yemeni refugees in the Netherlands to help fill this gap in evidence and help design potential integration and prevention strategies. In particular, the aim of this study is to explore the experiences of Yemeni refugees in the Netherlands with the Dutch healthcare system from a HL perspective.

## The HL framework

Sørensen et al. (2012) [2] developed a comprehensive HL framework containing four dimensions that function on three domains to define HL. The four dimensions are: *access*, *understand*, *appraise*, and *apply* [2]. First, *access* defines the capacity to look for and obtain health information. Second, *understand* describes the ability to realise and grasp health information. Third, *appraise* refers to the individual’s capacity to compare, explain and interpret health information. Fourth, *apply* describes the extent to which the individual can employ health information to make informed decisions and maintain a healthy lifestyle.

The processing of health information occurs in three domains. First, *healthcare* is about the ability of the individual to access, understand, appraise, and apply medical information to produce informed judgments on medical conditions and adhere to medical instructions [2]. Second, *disease prevention* describes the capacity of the individual to access, understand, appraise and apply health information on risk and protective factors[2]. Third, *health promotion* refers to the ability to access, understand, appraise, and apply information on the physical and social determinants of health to maintain a healthy lifestyle [2].

HL is also impacted by the distal and proximal factors that differ between people [2, 5]. Distal factors include social and environmental determinants such as demographic composition, language, political views, and culture [5]. For example, cultural barriers can discourage migrants from accessing mental health services because, in some cultures, mental healthcare is stigmatised [13, 18, 19]. Proximal factors include personal determinates and situational determinants. Personal determinants are gender, age, ethnicity, race, education, and socioeconomic status [5]. Situational determinants are family and peer pressure, social support, and the physical environment [2, 5]. It is relevant to consider these factors because migrants usually experience language and cultural barriers, family separation, and lower employment opportunities in the host countries [7, 12, 13].

## Methods

### Research design and setting

This study has an explorative nature with a qualitative phenomenological approach. Yemeni refugees in different parts of the Netherlands were the target group of this study. This approach is appropriate for exploring experiences and examining how meanings are ascribed to these experiences [20]. Results from the investigation were interpreted using the HL framework; adapted from Sørensen et al. (2012) [2]. The study was approved by the Ethics Review Committee Health, Medicine and Life Sciences at Maastricht University (FHML-REC/2020/004). Study participation was accepted after informed consent was signed.

### Sampling and sample size

Participants were approached using non-probability convenience and snowball sampling. Initially, five Yemeni refugees in the Netherlands – known to the research team from Yemeni cultural events due to the Yemeni origin of the first author – were invited to participate in this study. This group was then used to snowball and invite other participants. All participants were at least 18 years old, holding a refugee residence permit, and of Yemeni origin. Thirteen participants agreed to an interview, which is enough to reach data saturation, partially also due to the homogeneity of the study participants [21].

### Data collection instrument and participant validation

In-depth semi-structured interviews were conducted by the first author in Arabic – the mother tongue of the participants. An interview guide was created by the research team based on the HL framework and survey developed by Sørensen et al. (2012) and Sørensen et al. (2013), respectively [2, 5] (see Supplementary file 1). The interview guide was translated into Arabic by the first author who proceeded with the interviews.

Interviews were conducted via phone calls. Each phone call took approximately one hour. It was not possible to carry out the interviews face-to-face due to Covid-19 lockdown restrictions. The interviews were tape-recorded with permission from the participants and then transcribed ad verbatim from Arabic and translated to English. To minimise the possibility of error, the recorded audio was played again to double-check the transcripts. To assess trustworthiness, seven participants were chosen as independent auditors to listen to their taped interviews and approve the English transcripts.

### Data analysis

To analyse the interviews, a thematic analysis was conducted using Microsoft Word and Excel (version 2016). Software specific to qualitative analysis was not used due to the conservative number of participants [22]. Participants’ answers were deductively analysed using the HL framework [2, 23]. Specifically, themes and codes were developed based on that framework to analyse the interview transcripts using colours and numeric codes before creating the overall coding frame. The coding frame was developed using the thematic networks analysis as described by Attride-Stirling [23]. First, textual data from the transcripts was clustered by highlighting relevant extracts that form the basic themes regarding experiences, perceptions, and knowledge of the participants. Second, the basic themes identified in the transcripts were grouped under the relevant HL dimensions, which were titled according to the organising theme. Third, the organising themes were assigned to the relevant health domains which were denoted by the global theme. These final global themes were deductively defined in advance based on the HL framework retrieved from Sørensen et al. (2012) [2]. The first and last author discussed the categorisation, interpretation and abstraction of codes in order to ensure consensus and saturation for major themes. Once disagreements were resolved, the rest of the research team had the opportunity to comment on the coding tree presented below.

## Results

Thirteen Yemeni refugees participated in the study, of whom there were only three women. The study was initially intended to include 12 participants at the minimum. However, the thirteenth participant was invited to reach a balanced saturation within the female sub-group.

### Demographic characteristics of the participants

Detailed information on the socio-demographic characteristics of the participants can be found in Table 1. As shown in the table, most of the participants are 25 to 35 years old males, have postgraduate degrees, and have been in the Netherlands for at least four years.

### Coding frame and quotation book

Table 2 presents the coding frame of the interviews. The table shows how data were analysed using the thematic networks analysis technique (the coding three). For more verbatim quotations, please check coding matrix (Supplementary file 2).

**Table 1:** Demographic characteristics of the participants

Participant ID	Sex	Age range	Years in the Netherlands	Educational level
1	Male	25–35	4	Postgraduate
2	Male	25–35	5	Postgraduate
3	Male	25–35	6	Postgraduate
4	Male	18–25	4	Undergraduate
5	Female	25–35	2	Postgraduate
6	Male	35–45	5	Elementary school
7	Male	25–32	7	Undergraduate
8	Male	35–45	5	High school
9	Male	25–35	5	Postgraduate
10	Male	35–45	3	Postgraduate
11	Male	25–35	3	Postgraduate
12	Female	25–35	2.5	Undergraduate
13	Female	35–45	3	Postgraduate

**Table 2:** The coding frame

Global theme	Organising theme	Basic theme identified in the interviews	
Healthcare	Access	GPs and pharmacies are accessible. Helpless/mistrust towards the GPs/ cultural barrier.	
	Understand	Knowing how to use prescriptions. Language barriers with understanding the leaflets. Struggle with understanding health insurance.	
	Process/Appraise	Acknowledging that doctors are the main source of medical information.	
		Knowing when to seek care/ ability to distinguish between the symptoms. Understanding the restrictions on over prescribing medicines.	
	Apply	Knowing how to contact the emergency. Consuming the medicines as the doctor instructed.	
Disease prevention	Access	Harmful consequences of smoking, drinking and physical inactivity. Acknowledging the role of mental therapy.	
	Understand	Lack of knowing the physical health consequences of alcoholism. Insufficient knowledge about vaccinations. Postponing seeking mental therapy. Understanding early screening/full check-ups.	
	Process/Appraise	Knowing what screening to take.	
	Apply	Taking the necessary vaccines. Undergoing early screening. Quitting unhealthy habits.	
Health promotion	Access	Emphasis on a healthy balanced diet/ fruits and vegetables. Emphasis on organic food. Emphasis on exercising. Ability to manage and improve mental health. Understanding health-friendly neighbourhoods. Understating collective responsibility.	
	Understand	Inadequate understanding of food labelling. Importance of health at the workplace.	
	Process/Appraise	The impact of housing conditions on health. Discerning the stress of trauma, language barrier, lengthy asylum bureaucracy, unemployment and family separation.	
	Apply	Joining sports clubs/ sports lessons/ self exercising. Reading food labels to keep a healthy diet.	

### Healthcare

The participants showed no difficulty in getting and processing information to access the Dutch healthcare system. All of them appeared to know how to access and understand the Dutch gatekeeping system and pharmacies. They also knew how to call emergency services. However, the majority showed helplessness and mistrust towards GPs. Participants expressed that they felt that their GPs were hard to be convinced of their health problems. Several participants also expressed that the opinions of Dutch GPs made them feel hopeless and prevented them from seeking care unless it was a serious problem. Furthermore, participants felt that the Dutch GPs too frequently recommended over-the-counter “pain killers” while neglecting their real pain and injuries:

*I was suffering lower back pain and went to the GP, and he directly prescribed Paracetamol to me. […]I went to him again […] and he prescribed me a stronger pain killer […]. But I also asked him to do X-ray; […]. I had to pay upfront first. […]. And after I did the X-ray, he told me that I had a little bit of abnormal bone structure, and it subsides with some sessions with the physiotherapist. […] I almost lost the hope in the healthcare providers here. Participant 2*

Additionally, newly arrived participants expressed language barriers when communicating with healthcare workers. Some participants reported that communicating with the GPs through phone interpreters was sometimes challenging. Compared to face-to-face interpretation, explaining their private health problems through the phone interpreter made them slightly reserved, especially when the connection quality with the interpreter was poor. Some also indicated that they still faced difficulty understanding medical prescriptions and leaflets. Furthermore, some participants lacked a proper understanding of health insurance, which rendered them vulnerable to financial risks. A few of them had incurred considerable costs due to misunderstanding their insurance coverage. There was also a specific concern expressed by a female participant about insurance coverage for reproductive care. This participant had struggled to understand the health insurance system in general because there was no similar insurance system in Yemen. The way that maternity healthcare insurance worked in the Netherlands was surprising to her

*This has been a problem for me. As you know, we do not have health insurance policies in Yemen. I have been struggling to understand the deductibles and bills that I get after I see the specialist though I was told it is covered. Another striking point is the maternal healthcare insurance, I was not aware that if you do not take it at the beginning of the year, you will not be covered if you give a birth afterwards. Participant 12*.

Interestingly, one participant added an explanation as to why some Dutch medical doctors were not perceived as trustworthy by some of the Yemenis refugees. He indicated that there was a cultural barrier. Specifically, the participant stated that the Yemeni people did not tend to express their symptoms, especially women. According to him, medical doctors should rather seek and actively elicit more information when doing physical examinations of people from such a culture:

*[…] My point is that our culture is a bit different. We do not express everything in detail. As a doctor, you need to extract the information from some people. This is especially clear with the women from our culture; they do not express everything openly. Participant 7*

### Disease prevention

Almost all participants identified and understood health risks associated with unhealthy behaviours such as physical inactivity, smoking and excessive alcohol consumption. Many risk factors and associated health outcomes were identified by participants: physical and mental health problems due to lack of physical activity, such as obesity, heart disease, or mental health issues. Similarly, cancers - especially lung cancer - and liver disease were identified as consequences of smoking and drinking alcohol. A few participants also stated that they quit smoking because it was bad for their health. Despite knowing the health risks of physical inactivity, smoking and excessive alcohol consumption, few participants stated that they were not aware of physical diseases due to drinking alcohol. Nevertheless, these participants showed that they knew the risks of physical inactivity, smoking and mental effects of alcoholism.

The majority acknowledged the importance of early screening and check-ups. Few of them even explained that undergoing unnecessary screening may backfire because it could induce a mental burden. Furthermore, several participants emphasised the importance of undergoing screening and check-ups, especially when they were at risk. However, some participants also exhibited limited awareness about vaccination. Although they indicated that vaccines prevent diseases, they stated that they lacked knowledge about the way vaccines worked.

*I just hear about them. It is like: vaccinate yourself it is good for your health and protects you from disease. However, honestly, my knowledge about vaccination is very limited. […] Regarding the flu vaccine, I have never heard about it. Participant 4*

Furthermore, some of the participants were aware of the importance of seeking professional mental health therapy when suffering from issues such as depression. Some of them also stated that they were against stigmatising mental healthcare, which was common in Yemeni culture. However, the participants indicated that seeing a mental health therapist was the last resort. Furthermore, one of the participants indicated that she noticed a lack of mental health awareness in the asylum centres. She also indicated that, due to mental health stigma, many Yemenis would not consult a psychologist even if they were depressed.

*[…] Going to the mental therapist is stigmatised in our culture. Many Yemenis will not go to the psychologist because of this even if they are depressed. […] I noticed that there is a lack of mental health awareness in the asylum centres. I saw many people depressed because of boredom and long waiting. I think it would be useful if there are mental health awareness tailored to the people who come from cultures that stigmatise mental healthcare. Participant 12*

Besides realising the importance of screening, check-ups and vaccination, some participants identified when these preventative measures were applied to them. For instance, some participants stated that they had used the seasonal flu vaccine because they suffered from chronic diseases. Participant 12 reported that she and her siblings underwent frequent check-ups for their heart function due to genetic heart conditions in the family.

### Health promotion

Most of the participants emphasised that a balanced diet and regular exercise are crucial elements in maintaining a healthy body. In addition, reducing sugar intake, taking vitamin D supplements, eating organic food, and eating vegetables and fruits were particularly valued by participants. Yet, only a few of them were aware of food labels; the majority either did not understand or misunderstood food labels and confused nutrition facts with ingredients.

*[…] the person should stay healthy in terms of eating and exercising. Also, it is important to check your calorie and vitamin intake. You know that people who come from our region need to take care of their vitamin D intake here in the Northern hemisphere. Participant 11*

Although the participants emphasised individual responsibility in health promotion, the majority indicated the importance of collective or governmental responsibility. They stated that healthy choices were not often under the individual’s control. They also provided examples of how the government was responsible for ensuring the health of all citizens by supporting low-income people, salt reduction policies, and subsidising farmers to grow and produce healthy products.

Besides emphasising diet and exercise, some participants indicated that housing conditions had an impact on health. They mentioned the importance of cleanliness, sunlight, ventilation and fighting harmful insects/animals at home. Additionally, some participants highlighted the importance of health at the works place and living in health-friendly neighbourhoods. The participants indicated that the conditions at the workplace, physically and interpersonally, were important to ensure healthy and productive employees. The participants valued green areas and the accessible public playgrounds in their neighbourhoods.

Furthermore, the participants showed the ability to manage mental health problems and improve mental wellness. Their responses included religious practices, diet, exercise, and engagement with close people. Few responses pointed out the major factors behind the mental struggle of refugees in the Netherlands. For example, participants attributed stress to their traumatic experiences during their smuggling into Europe, lengthy asylum bureaucracy, the lack of work opportunities, language barriers and family separation. One participant stated that he had started smoking when he arrived in the Netherlands due to stress created by unemployment and language barriers.

## Discussion

The findings reveal the experiences of Yemeni refugees in the Netherlands with the healthcare system in the Netherlands and their health literacy. Participants were able to access, understand, judge, and apply information in the three HL domains that were analysed, namely, healthcare, disease prevention, and health promotion. However, there were some significant experiences and issues among this group.

First, the findings concerning language barriers during the first months after arrival, especially comprehension of medical leaflets and prescriptions, were in line with a study done among Ghanaian migrants in the Netherlands [24]. Language barriers become more pressing when migrants cannot speak Dutch and have no command of English [24, 25]. The Dutch government offers free phone interpretation for refugees [25]. However, some participants in this study indicated some interpretation services are only available by phone, which hinders sharing private information. Thus, the quality of cultural mediators and interpreters should be investigated and improved in the future. Medical leaflets for common diseases are actually translated into Arabic by the Dutch Municipal Health Service (GGD) [26]. However, the awareness of these translations might not be enough among newly arrived refugees who are often preoccupied with other matters.

Second, almost half of the participants expressed a lack of understanding of health insurance schemes. This has been found previously among Ghanaian migrants in the Netherlands [24], which can be ascribed to differences in healthcare system structure compared to the country of origin. For example, almost all people in Yemen are uninsured, and the healthcare system is mostly based on out-of-pocket payments [27]. Furthermore, migrants experience a difficult shift from readily available healthcare at the asylum stage to having to navigate through the healthcare system themselves [28]. Therefore, they struggle to understand health insurance schemes, especially in insurance-based systems where regulations are often more complicated than in tax-based systems [18].

Third, participants’ proclamation of mistrust and helplessness towards GPs was striking, but reported among other ethnic migrant groups in the Netherlands as well. For instance, a majority of Somali migrants feel that they are not taken seriously by GPs, are over-prescribed pain relievers and are refused referral to specialists [29]. They also indicated that they are confronted with a generalising attitude by some GPs due to previous experiences with demanding patients from the same ethnic group [29]. Hence, some of them dislike visiting Dutch GPs and prefer to be treated in neighbouring countries [29]. Similarly, Ghanaian migrants in the Netherlands think that GPs are reluctant to prescribe them the medication they need, and some even expressed dissatisfaction with the lack of expertise of doctors [24].

A reason for this undermined trust is the gatekeeping role of GPs in the Netherlands and the specific culture the migrants belong to [24, 29, 30]. For instance, Middle Eastern cultures – including the Yemeni one – have different unique trust norms. These cultures are generally characterised by strong involvement with close social networks, such as family and friends, and with distrust for people considered outside of these networks [30]. Illness perceptions of Yemeni people also have strong cultural links to traditional medicine and religion [31, 32]. Therefore, it is paramount for GPs to first establish trust with migrant patients in order for them to integrate better into the Dutch healthcare system. This finding is in line with several studies done on the cultural competence of Dutch healthcare providers [29, 33, 34]. Furthermore, there are also cultural differences in communication styles and several studies have confirmed that a GPs communication style matters when treating patients with migrant backgrounds [35, 36]. Patients from individualistic cultures tend to possess a more assertive and expressive communication style [37]. However, patients from collective cultures, such as the Yemeni one, usually have a communication style characterised by respect for authority and indirectness [37]. Particularly, people from Middle Eastern cultures exhibit passivity in the presence of medical doctors (authority figures) and do not question their input [30]. This lack of expressiveness among Yemeni people was observed in this study, especially among women.

To address this mistrust, competent patient-centred and intercultural communication to improve trust, health outcomes, patient satisfaction, and enhance physician satisfaction is worthwhile [35, 36, 38]. Previous studies found that medical doctors in the Netherlands lack some important intercultural communication skills, such as being aware of cultural expectations, using patients’ relatives in decision making, and checking patient’s language ability [38–40]. In fact, this type of training is underemphasised in the medical curriculum in the Netherlands [38], and in general, cultural competence teaching should be made more explicit in curricula [41]. Berry (2007) also highlights that physicians should not treat people from the same culture as a homogeneous group due to the differential effect of gender, education level and beliefs [37].

Fourth, participants’ lack of knowledge of vaccines was explored by De Vito et al. (2019), who observed that educative materials on vaccination are scant in migrants’ native languages [42]. Other studies show that vaccination rates are very low in the countries of origin and/or migrants have lower vaccination updates in Europe compared to the natives [43, 44]. To tackle this, door-to-door initiatives to screen for high-risk migrants, issue immunisation cards according to countries of origin and host countries, and disseminate materials in native languages about vaccination are effective strategies [42, 44].

Furthermore, postponing seeking mental health treatment by participants, despite experiencing severe depression, is consistent with previous studies [19, 42]. A possible reason for this phenomenon is that migrants and refugees are not fully aware of the importance of mental health and their mental healthcare entitlements [45]. Involving ethnic mental health therapists is an effective strategy to resolve this because it makes seeking treatment more culturally acceptable [19]. For example, culturally tailored cognitive therapy with bilingual therapists or therapy in the migrant’s preferred language was proven to reduce depressive symptomatology [46]. Additionally, participants misunderstanding food labels raises the question of whether their knowledge of a healthy diet is adequate to maintain a healthy lifestyle.

### Strengths and limitations

This study provides a unique attempt to explore the HL and experiences of Yemeni refugees in the Dutch healthcare system. It also highlights potential pathways for inequalities between native populations and migrants or refugee groups that can be helpful to policymakers and researchers. For instance, addressing migrants’ and refugees’ undermined trust in healthcare providers is relevant since our findings are highly comparable to those found among refugees in Sweden and Somali and Ghanaian migrants in the Netherlands [13, 24, 29]. However, there are some limitations. First, nearly all participants have higher education degrees which may bias the results since it correlates with higher HL levels [47]. Second, this study focuses only on Yemeni refugees while there are bigger migrant and refugee groups coming to the Netherlands, such as Syrians [10]. Hence, it is worthwhile to study the experiences and HL among other migrant groups to have a more holistic view. Third, due to the small sample size and its qualitative nature, the representativeness of our results is uncertain. Nevertheless, given the homogeneity of participants, it still offers valuable in-depth insights on the topic. Analysis of collected data was also done manually, although, for the conservative number of participants, the use of software was not necessary [22]. Fourth, although some participants approved of the trustworthiness of their translated answers, the translation of the interview guide into Arabic was done by a single person. Finally, the cost-effectiveness of recommended interventions in this study requires future investigation.

## Conclusion

Overall, Yemeni refugees in the Netherlands seem well-acquainted with many aspects of health literacy: they can access, understand, appraise, and apply health information in the domains of healthcare, disease prevention, and health promotion. However, some refugees lack an understanding of health insurance schemes, vaccination, and food labels, and experience language barriers after arrival. Furthermore, they are inclined to postpone seeking mental healthcare. They also showed mistrust towards GPs and perceived them as uncaring and hard to convince of their health complaints. To address these issues, cultural mediation services available for migrants and refugees require improvement, and training focused on understanding cultural-diversity, developing cultural competence and intercultural communication may benefit healthcare providers. However, due to the qualitative nature of our study, the findings cannot be generalized. Large-scale quantitative research is therefore needed to examine HL and experiences among refugees in the Netherlands with the healthcare system. This is crucial to prevent health inequalities and to tackle unmet health needs regarding mental healthcare, access to primary care and vaccination.

## Electronic supplementary material

Below is the link to the electronic supplementary material.

Supplementary Material 1

Supplementary Material 2

## Data Availability

The datasets used and/or analysed during the current study available from the corresponding author on reasonable request.

## Abbreviations

HL: Health Literacy
GP: General Practitioner
FHML-REC: Ethics Review Committee Health, Medicine and Life Sciences
