# Supplementary material for: Yemeni refugees’ health literacy and experience with the Dutch healthcare system: a qualitative study
Source: BMC Public Health. 2023 May 18;23:902. doi: 10.1186/s12889-023-15732-6 (PMC10193334; doi:10.1186/s12889-023-15732-6)
Supplement: Supplementary file 1 — Supplementary Material 1 [file 12889_2023_15732_MOESM1_ESM.docx]

The interview guide:

| Interview guide [adapted from Sørensen et al. (2013) (5), the interviews were in Arabic]:  1] The first domain: healthcare:  Tell me about your ability to manage medical issues and information?  Dimensions: access, understand, appraise and apply [AUAA].  Key terms for follow-up questions:  Information about symptoms of illnesses, treatments of illnesses, what the doctor says, understand the leaflets, medical emergency and call an ambulance, follow the instructions, is what in the media reliable, advantages and disadvantages of different treatment options.  2] The second domain: diseases prevention:  What do you think about disease prevention?  Dimensions: AUAA.  Key terms for follow-up questions:  Information about managing unhealthy behaviours and mental health problems. Understand health warnings about behaviours, vaccinations, health screenings, know when to go to doctor check-up, know how to protect yourself from illness based on information.  3] The third domain: health promotion:  What do you think about: “health should be promoted by the individuals and the community to create much healthier environment and community”?  Dimensions: AUAA.  Key terms for follow-up questions:  Information about exercise and diet, food packaging, mental well-being, health-friendly neighbourhood, political changes that may affect health, health at work, housing conditions and health, joining sports club or exercise class. |
| --- |
