# Supplementary material for: Yemeni refugees’ health literacy and experience with the Dutch healthcare system: a qualitative study
Source: BMC Public Health. 2023 May 18;23:902. doi: 10.1186/s12889-023-15732-6 (PMC10193334; doi:10.1186/s12889-023-15732-6)
Supplement: Supplementary file 2 — Supplementary Material 2 [file 12889_2023_15732_MOESM2_ESM.pdf]

Table 1: color labels

Themes	Codes
Healthcare	Access
	Understand
	Process
	Apply
Disease prevention	Access
	Understand
	Process
	Apply
Health promotion	Access
	Understand
	Process
	Apply

Table 2: extract matrix

Themes	Codes	Basic themes from the interviews (quotes)
Healthcare	Access	"I call my GP if the problem is serious." P1
		"[…] according to the system here, I have to go to the GP when I am suffering a pain or painful symptoms." P2
		I think it is not difficult to make the appointment but I had a story about the GP here. I was suffering lower back pain and went to the GP, and he directly prescribed Paracetamol to me. I do not think he was asking me sufficiently about what I do and how I ended up here. He just told me it is normal and people just suffer from it at some point of their life. I really did not trust what he was saying especially when the pain kept increasing over time. I went to him again and asked him that the Paracetamol does not work and he prescribed me stronger pain killer (I think it was called Ibuprofen). But I also asked him to do X-ray; though he said to me I am not going to authorize it. I had to pay upfront first. I said it is ok. And after I did the X-ray, he told me that I had a little bit of abnormal bone structure and it subside with some sessions with the physiotherapist. "P2
		I almost lost the hope in the healthcare providers here. P2
		I will generally make an appointment with the GP. But it is hard sometimes because sometimes you need to go directly to the hospital. I once had very high fever and just went to the hospital. P3
		it is fairly easy but sometimes the GP does not let you go to the hospital or the specialist which makes you feel helpless going to them. […] Regarding the pharmacy, overall it is pretty easy to go to the pharmacy after your doctor has issued your prescription and the pharmacists show you how to use the drug. P3
		in the Netherlands, you have to have a family doctor. This doctor keeps a history of you and you should consult him when you have health problems. […] Another important point is that going to the doctor here is different from Yemen. In Yemen, you will have a lot of drugs and injections for normal fever. However, the doctor in the Netherlands will normally prescribe you Paracetamol and make your immune system fights for you. However, this can be annoyed sometimes especially when you go to the doctor for a abnormal pain, he will try to calm you and prescribe you Paracetamol. Personally, I was suffering from abdominal discomfort and every time I went to the doctor, he will prescribe pain killers. After year and a half, they figured out that I have allergies from milk products. This may make you feel helpless to go to the doctor. P4
		[…] you would go to your doctor. Here, people here have a doctor they go to when they have symptoms of a disease. This doctor is responsible for evaluating your case: if it is manageable then he will treat you; otherwise, he will refer you to the specialist or hospital. […] P5
		[…] In terms of making the appointment, this is an easy process. With regard to being with your doctor, you have to exaggerate your symptoms to go to the specialist or the hospital. […] Personally, I do not feel really confident being with the family doctor here. Sometimes he looks up my symptoms in the computer which concerns me whether he is really able to know what I am suffering from. For example, I cannot use my hand to catch things and write and it really hurts me sometimes. I have been going to my doctor but every time, he tries to make the problem psychological. This really annoys me. I do not go to the doctor for nothing. I usually go to the doctor when the problem is really unbearable. P5
		[…] I will go to the family doctor. This doctor usually prescribes you Paracetamol no matter what. […] I think that medicine here is overrated. It is true that they stimulate your immunity to work but sometimes it is too much. […] This is the reason why I do not like to go to the family doctor unless I have severe symptoms. P6
		Firstly I will contact the family doctor and he decides on my case if I need to go to the hospital or to the specialist. P7
		if it is really bad problem, I will call the emergency. […] In the normal situation, I will call the family doctor and ask for an appointment. P8
		seeing a specialist is a bit annoying process sometimes. While we have been referred by our family doctor to specialists, the specialists oftentimes will say similar things while charging us extra money. This happened to my son, with me and with my wife. My son has otitis and we went few times to specialists but ended up with nothing major added to what the family doctor said. At the same time, we have to pay extra fees. Also, me and my wife went to a specialist to check some nerve problems but ended up in paying the own risk twice in one year without us knowing this financial risk properly. Another annoying point is the dentists, they are not clear when you ask them how much is covered by my insurance. I had a teeth problem few times and when I asked the dental clinics about the costs before doing any intervention, they will make everything looks covered by my insurance. But then I got extra bills to pay week after these incidents. It is really annoying. This has cost us a lot of money with us being poorly informed in how exactly the health insurance works. This was worse before my family moved to the Netherlands and I had to do the communication and insurance arrangements with the doctors myself. It was difficult especially my Dutch is poor and I had to accept what they say. P8
		[…]The first thing to do here is contact the family doctor. If he is unable to address the problem, then he will refer you to the hospital or the specialist. […] P9
		if the problem is not serious, I would stay at home and take rest. But if it persists or I feel the symptoms are serious that my immunity is overwhelmed, I will directly make an appointment with the family doctor. P10
		[…] The first step is you should call the family doctor and make an appointment. The family doctor then decides whether if your case is manageable; otherwise he will refer you to the hospital or the specialist. P11
		[…] I call my family doctor to make an appointment. After that, he will determine if I need further help. But I do not go every time to the doctor. If I feel a normal cold, I would stay home. P12
		[…] I will try to address the condition myself because I studied medicine. So I would take pain killers and rest my body if the symptoms are a manageable; otherwise, I will call the GP. P12
		Important point, which I find it problematic here, is meeting the expectation of the patient. I had to undergo a deviated nose surgery in the Netherlands and it was useless. As a doctor, I know that this operation is not very difficult given the technology that we have nowadays. However, this is not the most annoying part of the story; I was upset when the doctor coldly told me that we will do the operation again. I felt like I am a testing sample. P13
	Understand	"yes. I have gone to both. […] No, the communication goes fine as my Dutch and English are fluent. Plus, going to them is essentially not difficult. You just have to make an appointment." P1
		"[…] I think the health insurance is a little bit complicated issue. I have suffered a lot of pain in my teeth and the doctor told me I had to pull out one tooth and I had to pay over 300 Euros as own risk (deductible). I was not aware of this and I think that this can be a big issue for some low-income people. People should clearly know what they expect and what they have to pay." P1
		I am fairly fluent in Dutch so no major problems here and in Germany my friend, who speaks fluent German, was with me. P2
		[…] communication wise, there is no problems as I speak Dutch and English. But I feel like this system […the GP gate-keeping system…] may miss the right diagnosis when sometimes takes too much time and leaving people on the Paracetamol. P3
		if I have a mild fever or cold, I will stay at home. But if I have very severe fever or other conditions, I will go to the doctor. P4
		No I do not have communication problems. I also speak Dutch fluently. […] Going to the pharmacy/pharmacist is not difficult at all. I have been to a pharmacy/pharmacist. I found my prescription ready to pick up and they showed how to use it. It was pretty easy. P4
		[…] going to the pharmacy/pharmacist is not difficult. However, the prescription are in Dutch and my Dutch is not good yet. I just settled my status here. Important point is that the pharmacists have not informed me about the side effects of the drugs I use. P5
		: […] no it is easy; you just need to make an appointment and go. The same also goes with pharmacy/pharmacist, you go there and you find your prescription already with the pharmacist and you just pick it up. Regarding communication, I use to have an interpreter with me but now I have my kids helping me. But I have faced problems with dentists. They scammed many of us who have oral problems. We usually go there and have our insurance with us and they tell us at the clinic that everything is covered regarding money. But we go home and receive insurance bills in few days. It is annoying and we cannot do anything about it especially in the first months of our arrival here. I believe that this is mainly because we do not know the language and the regulations properly. P6
		it is simple and easy; you just need to tell the pharmacist that your prescription is on the system and they will check it and inform you how it should be consumed. P7
		I think that the family doctor is very good idea. I mean that you have a doctor who knows your history every time when you go to him. I have not had this when I was in Yemen. But this is probably due to the fact that the Yemeni funds towards health are poor. […] Another issue - which is related to the personality of the doctor here in the Netherlands – is how he deals with people from other cultures. For example, during my early months here, my Dutch was poor and I felt that some doctors were not asking the questions about the problem that I am trying to communicate to them. I felt that few of them they know how to ask questions about your problem. However, I felt some of them were trying to just quickly diagnose you with minor things and prescribe you Paracetamol so they stay in the safe side. […] My point is that our culture is a bit different that we do not express everything in detail. As a doctor you need to extract the information from some people. This is especially clear with the women from our culture; they do not express everything openly. […] P7
		[…] In terms of going to the doctor and making appointments, it is easy. […] I have my sons – who are fluent in Dutch - helping me with that as well. The same also goes to the pharmacy/pharmacist. I always go there and receive my prescription without any problems. P8
		[…] it is easy to get to the family doctor. You just need to call them and make an appointment. […] Communication wise, I have no problems because I speak good Dutch and fluent English. P9
		[…] Regarding the pharmacy/pharmacist, it is convenient because everything is digital here. When your doctor tells you that your prescription is ready, you find it already in the pharmacy's digital system. In addition to the prescription, the pharmacist also shows you how it should be used. […] However, it can be a problem if your Dutch is not that good; so you always need to be careful with the prescription, the doses and side effects. P9
		[…] Yes, I have a problem understanding the health insurance. I tired really hard to understand the premiums this Year so I avoid the last year extra bills from my dentist. I increased the premium for the oral health and made sure that it is going to cover my needs. I went to the dental clinic and ended up receiving extra bill. It is just disappointing; a lot of information for the premiums and it seems unclear and misleading for me. P9
		[…] Going to the GP is easy. His office is in the neighborhood. You just need to call them, explain your problem and make an appointment. […] It is also easy to deal with pharmacies. After you receive your prescription from the doctor, tell the pharmacist your day of birth and he will find it in the system.P10
		Communication wise, it can be problematic sometimes to explain everything in Dutch because my Dutch is not that good. However, I always manage with my English. P10
		This is not the problem; the problem is when the doctor does not seem convinced about your problem. I am personally a pharmacist; I studied pharmacy in Yemen. So I know quite well about the drugs and some aspects of the disease. The problem is that my son once had one of his ears hurts and caused him fever. So we took him to the GP. I tried to tell the doctor that this looks like an ear infection symptoms so it might need antibiotics. She was not convinced even after she saw some pus coming from the ear. She said stay for at least two days to see how the prognosis goes. But after three days, my son starts losing a bit of his hearing which made me afraid that the pathogens may also get into the brain and make everything worse. Even after all of this hassle we could not get antibiotics but fortunately the problem started subsiding thereafter. […] I understand and agree that over prescribing antibiotics is really dangerous. However, this does not mean you become too careful that you under-treat people. P10
		Yes, it was complicated for me to understand the health insurance at beginning. You know that we do not have health insurance system in Yemen. So I spent a lot of time reading and asking about different providers, basic insurance, different premiums, dental care and own risk. P10
		[…] I have not had a communication problem with the doctors because, besides Dutch, I speak English well. But I also many people who struggle with the doctors because of the language. […] With regard to going pharmacy, it is simple and easy. When you go to the pharmacy, your prescription is already placed at pharmacy's system by your doctor. You just need to pick it up.
		The family doctor can be problematic sometimes this especially when I was residing at the asylum center. It was almost impossible to get a referral from the family doctor at the asylum center to the hospital. But after I got out the center, it relatively easier but it still depends on your family doctor. The family doctors will often tell you that you are ok and will give you Paracetamol. P11
		[…] so far, going to the family doctor is simple and easy. The same also goes with going to the pharmacy because your prescription is already in the system after you leave your doctor. […] I do not face major problems in communicating with the healthcare providers because I can speak English very fluently. P12
		[…] Though I usually receive sufficient instructions from the doctor and the pharmacist in how to use drugs. But this can be difficult sometimes if you miss something because everything is in Dutch. It is not only Dutch; there are some special terms that I cannot understand. So I look up the internet sometimes, for example, to check the side effects. […] It can be helpful if the perceptions and the information about the drugs is available in English especially for the newly arrivals migrants. P12
		I have been as an interpreter for some residents at my asylum center before I left it. I noticed that some people find it really difficult to communicate with the doctors in the asylum centers. This is because the phone interpretation service can be bad sometimes and some people become a bit reserved to explain their private problems in the phone compared to face-to-interpretation. P12
		[…] This has been problem for me. As you know, we do not have health insurance policies in Yemen. I have been struggling to understand the deductibles and bills that I get after I see the specialist though I was told it is covered. Another striking point is the maternal healthcare insurance, I was not aware that if you do not take it at the beginning of the year, you will not be covered if you give a birth afterwards. P12
		[…] I have not faced major problems with going to the GP. However, I had a language barrier with some doctors during my first months after arrival but I was able to manage with my English. Though it was not a big problem for me, I saw some refugees really struggle with it. P12
		[…] I have been to a pharmacy. It is easy to go there after you get your prescription from the doctor. The pharmacists are also very approachable. P13
	Process	"yes; if it is normal fever or cough, then I usually stay home, take rest and treat myself. But if it takes much time or very painful symptoms, I will go to the doctor." P1
		"I go over the internet and see if there is a safe recipes that I can do at home. I usually do this if I have abdominal discomfort; I will mix some natural herbs and it usually helps. But If it is feels painful and serious, I will go to the doctor." P1
		. If I have a common fever or cold, I would just stay home. But when it is a bit too annoying, I will call the GP but if it is too much, I will go to the hospital. P3
		I feel like the role of the doctor is complementary in some instances. I think everyone should take care of their health. But this does not mean you exclude the expertise of the doctor who studied medicine for years. […] I mean it is the general health literacy like using the internet and reading books about health. For example, now I am on low carbohydrate diet because I read and followed science series about the harmful effects of over-eating sugar. P3
		[…] I use the internet. I found YouTube very helpful. I got some insights for my allergies from milk products. P4
		Generally, you would go to your doctor when you really feel the symptoms. For example, I won't go to the doctor if I have cold. P5
		[…] I usually check the internet. […] However, I still believe that doctors are main source for medical information. P5
		I used to look up the internet but now I am familiar with the situation here. Currently, I check the official family doctors' website in the Netherlands to see how much of a help I need in certain medical issues. […] I do not fully trust whatever written in the internet. There is a massive amount of information over there. I once had a skin allergy on my hand and the internet confused and upset me as it showed me it could be a cancer. Then after consulting the doctor, it turned out to be a normal weather-related condition. P7
		[…] sometimes I see things in the internet. Or sometimes I call my doctor friends in Germany. […] This is in addition to going to the doctors here. P8
		[…] I always go when I feel really bad and the symptoms does not subside for days. It is also because I got used to response from the family doctor. He will probably give me Paracetamol which I have at home. It is good because doctors here want to make our bodies defend against the diseases. P9
		[…] I look up online. […] But this does not mean I look up everything. I only do the things that are natural and will not have serious side effects. For Instance, I would check the internet for the honey and herbal recipes for sore throat. Also, I sometimes ask my friends for the things that they tried and find them effective. This is especially for the minor health issues of my children. For example, if my children have a cough then I will call some other families that I know about the effectiveness of the common cough syrups. P9
		[…] My daughter once had a persistent fever and we were told to give her Paracetamol but not exactly how. We were giving her the dose when the temperature goes up. But after few nights the temperature went really high and my daughter displayed kind of epilepsy. We rushed to the emergency room and it turned out to be a "fever convulsion". It was all due to our misunderstanding of the way we administer Paracetamol to her. We were told that it is wrong to wait until the temperature goes up, we should have given it to her like every 6 hours. P9
		[…] I am pharmacist myself and I have worked in pharmaceuticals for 6 years in Yemen. So I know some aspects of health and disease. I also check some official medical websites in the Netherlands to see when I should go to the GP and when to go to the hospital and other medical information. P10
		[…] Usually, I do not call the family doctor if I feel my body can cope with it. It is because, as I said, 90% you will get Paracetamol. So I would check the internet for these manageable problems. P11
		[…] I check the internet which is not always good because there are a lot of information and you always need to be careful. I usually use the internet for normal stuff like the healthy lifestyle, herbal and alternative medicine. I notice that when you go over the internet for everything, you become more worried because the available information provide all extreme possibilities for your symptoms. So you need always to call a doctor because it is their profession and they know how to approach the problem. We also call my husband's relative to hear from him about medical issues because he is a doctor. P12
	Apply	"I will call 112. I already called the emergency for my friend who stopped moving his body after his sugar level went really high. I called them and they came quickly." P1
		"I followed everything literally and regarding the pharmacy thing. […] yes I went for my prescription and it was easy."P2
		yes I know it is 112. P3
		[…] I will call the ambulance. […] It is 112. P4
		I will call 112. P5
		yes, it is 112 P7
		I call the ambulance or the emergency in the hospital. […] I have the phone numbers of the ambulance, the emergency for our hospital and the family doctor saved in my phone. P8
		[…] I will call the emergency. […] It is 112 to call for the ambulance. P9
		I will directly call 112 to get the ambulance. P10
		I should contact 112 to get the ambulance.P11
		: […] I will contact the ambulance. […] The emergency phone number is 112. P12
		I will call the ambulance P13
Disease prevention	Access	"[…] certainly. It is like the Corona quarantine that we have these days." P1
		"[…] you are just bringing the disease to yourself when doing these things. It also makes your life so stressful. […] Exercising will definitely make your body feel better physically and mentally." P1
		[…] I think this is true' if someone does not exercise and eat properly he will end having bad diseases that can be avoided at the first place. P2
		"he will be prone to obesity and mental problems." P2
		it negatively affects the lungs and the heart. I think it makes some sort of plaque inside your lung and heart which inhibits the lung and the heart function. P2
		I think what we eat is very important when it comes to prevention. As I told you I followed this series about diet and sugars and now I am convinced that going on balanced diet, and avoid too much sugar and replace them with fruits. This will prevent a lot of physical and mental problems. I still have this in mind which is really interesting that over eating sugar is like over fueling your brain which in return disturb your mental health. P3
		[…] True, you should protect yourself before you get the diseases. For example, in this coronavirus situation, I should not leave my home as I do normally and cannot travel to my friends. By doing so, I am trying to prevent the disease to catch me and to prevent transmitting it to others. These are preventive measures. P4
		they are all unhealthy behaviors. Physical activity is important for you to concentrate, be active and be mentally relaxed. Regarding drinking, I think it affects your brain so much and causes addiction. But I do not specifically know what are the physical diseases caused by drinking. Concerning smoking, it can cause you cancer and heart diseases. P4
		You should try to eat fruits, vegetables, generally keeping health diet and walk/exercise daily. Also you should report your health problems to the doctor as soon as you feel they are unburnable. This will help preventing chronic diseases. […] For example, if you feel occasional pain from your kidneys' side, you should check it before it becomes a kidney failure. P5
		Physcial inacivity can cause overweight, bad cholesterol, body weakness and makes you more prone to heart diseases. Regarding smoking, it causes lung cancer. I think drinking also is very harmful for your liver. P5
		[…] I believe that smoking and drinking are harmful for people. First of all I do not drink that much but I smoke and I feel smoking is a treatment for me in some way. I have faced very helpless and depressing days when I first arrived in the Netherlands. I have stayed for 15 months without having my asylum application settled. This affects me so much in terms of language and work opportunities. So at that point I started smoking and felt smoking clears the negative energy from my mind. […] P6
		Regarding physical activity, It depends on the person. Personally, I do not have enough time because I have 4 children but I walk a lot. […] I also do not sit a lot because it stresses me. I like to go out and walk for hours. I think this is enough exercise for me. I also go with my kids to the football ground and encourage them to play. But I do not see myself able to do any vigorous physical activity at this point; my body is not prepared for these activities anymore. P6
		: […] this is very important; it perfectly applies to the current Coroanvirus situation. Social distancing embodies the "prevention is better than the cure". P7
		[…] I believe that they are harmful. They negatively affect the person physically and mentally. […] They can cause heart diseases, lung diseases, diabetes, cancers, obesity and mental issues. However, I do not how they really cause the disease physically especially alcohol. […] I myself was smoking in the past. But fortunately I quit. It was all because of stress but I think when replace this habit with healthy behavior, it will disappear. P7
		Look at the Coronavirs situation now; if we are not taking the distancing measures, we will be at massive risk. P8
		[…] these practices basically kill you. […] When I exercise and walk, the sugar levels goes very normal in my blood. […] Smoking is expensive and makes you an addict which brings you family troubles. I was smoker myself but fortunately I have quit. P8
		[…] When you keep yourself clean and eat healthy you basically prevent a lot of diseases. P9
		…] These behaviors have negative impacts on your health. I believe that physical activity boots immunity. […] The physical inactivity causes obesity which leads to diabetes. […] Smoking can harm the respiratory system and causes heart and lung cancer. […] Regarding drinking alcohol, I do not exactly know the diseases that are caused by drinking except for intoxication. P9
		[…] this is definitely the best. […] You need to stay healthy: eat healthy, exercise and sleep well. P10
		[…] Fortunately I do not any of them. Drinking, for example, is addictive and causes liver cirrhosis. Smoking is harmful too. It can cause all types of cancer and it is waste of money. […] Physical inactivity makes you relatively prone to all different diseases because your lifestyle is basically unhealthy. I remember reading about an American company which offered a break for exercising during the working hours. This resulted in a substantial cut in the healthcare allowances thereafter. P10
		[…] indeed, I agree. I believe we should eat healthy and keep healthy sleep habits so we avoid diseases. P11
		[…] I believe they are dangerous to your health. Lucky me that I do not do any of them. […] People who are physically inactive are prone to overweight, higher cholesterol levels and hypertension.P11
		[…] it is one of the most important roles in life. I try very hard with my family to stay healthy. For instance, we try to obey the social distancing rules to prevent the spread of Coroanvirus. P12
		[…] If you eat and do not exercise, you will be more vulnerable to diabetes, hypertension and mental problems. Regarding smoking and drinking we do not do them. I used to smoke but I quit before I moved to the Netherlands. As you know, they are harmful to the lungs and kidneys. […] But I do not exactly know what kind of specific diseases caused by smoking except for the respiratory problems in general. Also I think that drinking a lot of alcohol may harm the kidneys and cause brain damage . But I am not sure. P12
		: This is very true. As you may know how they maintain the primary healthcare here in the Netherlands. It is powerful method of prevention. Also, another way of prevention is when the chronic patients do regular checkups so they prevent any complications. P13
		[…] as a lifestyle, they are surely very harmful physically and mentally and that why the government here encourages exercising and taxes tobacco and alcoholic products. P13
	Understand	" […] I have no full view on this. But I believe we have to take the vaccines so we would completely avoid the disease. However, my information on this are insufficient." P1
		"[…] I agree with this. I used to do it yearly; not in Yemen but when I left Yemen to live in another country for my studies. I did it once in Netherlands but it was not full body check up. But I think this really helps to identify any diseases early on especially our diets are not good, I do not exercise regularly and I smoke." P1
		we had it back home in Yemen but never know how it really works except for the fact it prevents the disease. But I do not know how. I also believe it is mandatory here in the Netherlands. But honestly I cannot answer you with more than this regarding vaccination. P2
		[…] I do not smoke cigarettes but I smoke hokkah. I think hokkah is not as addictive as cigarettes. People who smoke cigarettes smoke everywhere and every time but at least for me I smoke hokkah few times day and sometimes a week. The smell of cigarettes is also disgusting. However, I am not promoting this view. I still believe they are both dangerous to health. P2
		very important to keep yourself healthy. People who do not sport at all are usually in a vicious circle of over eating, being overweight, feeling depressed then resorting on over eating again to alleviate the mental instability. P3
		I think they are part of the "prevention is better than the cure"; we can avoid diseases before we have them. But I do not know how vaccination work. I never had a vaccine when I am adult. However, again, diet and healthy life style are paramount for me. P3
		[…] I just hear about them. It is like vaccinate yourself it is good for your health and protect yourself from disease. However, honestly, my knowledge about vaccination is very limited. […] Regarding the flu vaccine, I have never heard about it. P4
		I think they are important to protect you from infectious diseases. […] Vaccines are a parts of the viruses injected to us so when we face them again, our body recognizes them and is prepared to react. But I have never used flu vaccine but I know a friend who did it. P5
		: […] I have never vaccinated my children in Yemen. I believe that you should depend on your natural immune system. My oldest kid has never received a vaccine before in Yemen and has never suffered a measure infection or disease. But now, I vaccinate them here in the Netherlands. You have to do it here in the Netherlands. […] Regarding the seasonal flu vaccine, I never heard about this and I never have had a vaccine in my life. P6
		I think that they are important and I vaccinate my child. […] It is a way of injecting you with part of the disease so when it attacks you in the future, your body is already familiar with it and can defend against it easily. […] It is also surprising to me that some people in the Netherlands are anti-vaccination. […] Regarding the flu vaccine, I have never had it. However, once I want to travel to Saudi Arabia, I took some vaccines requested by the Saudi government. P7
		[…] I believe that they very necessary. You can protect yourself from some diseases and viruses after taking the vaccine such polio vaccine. My brother has some thoughts against vaccination but I believe that their advantages outweigh their disadvantages. Also, I have taken the seasonal flu vaccine once because I have a chronic disease (…diabetes). P8
		[…] I think it is useful. To my knowledge, you undergo a checkup for certain diseases so you identity them earlier and prevent yourself from worst scansions. […] However, I do not know when you should do it. Maybe if the person suffer from symptoms of a certain disease, then he should do it. P9
		[…] I think they are amazing and I hope we find one for the Coronavirus. I know that the Dutch government has a basic free package of vaccines like polio vaccine. When you have kids, they will go on a schedule for these vaccines. P10
		[…] well, I am very careful with this topic. Though many people are against vaccination, I have been committed to it since I was in Yemen. […] It is because I believe that vaccines can prevent a lot of diseases. This also goes with making full use of all available means first, and only then leave the final result to God. […] I personally used to take the seasonal flu vaccine regularly when I was in Yemen. P11
		[…] so far I think they are important because they prevent you from getting serious diseases like measles. […] I actually vaccinate my son; I have done all of the recommended vaccines. […] They are basically a part of the disease given to the person so the body can learn and create an immunity against the disease. […] Yes, I have heard about the flu shot, but I never had it before; though I will take it if I am at risk. P12
	Process	"no. I never heard about this. But if it is necessary I will take it." P1
		I think this is very important. I also have a chance to do it once a year with my current insurance scheme. I think the person can check if he has diabetes early on especially when he eats sugar all the time. So he can prevent worse scenarios. This can also show if you have cancer. P2
		[…] things like full checks ups are important to regularly check if you have any diseases early on especially the chronic ones. If I have the resources to do it, I would do it every year. P3
		[…] it is very good to know if you are at risk of dangerous diseases. But I do not agree with making full checkups. You may end up knowing diseases that are going to disappear over time; without treating them. This may causes you stress and you just add problems to yourself out of nothing. P4
		It is extremely important especially against cancer. I know some women who did it and avoided late stages of breast cancer and others who did not and ended up in very extreme treatment regimens. P5
		[…] just trust your body, be happy and be satisfied with your life. Screening may reveal something that will stress me out and then I will end up developing the disease because of stress. I believe that the body can treat itself over time and if it is your fate, only then treat it. P6
		[…] I really like this idea. For example, if there is a certain type of genetic cancer or disease in your family, you should do it so you can avoid worse consequences. And this also a reason why I mentioned earlier that it is effective that we have family doctors in the Netherlands that they know your history very well and can make links when you have a problem. P7
		Everyone should go healthy before getting the problem. […] The healthy diet is key in this part. For example, if you eat everything without consideration you might end up like me having diabetes. […] I would usually go organic diet and products that are n
		:[…] I agree with this. Everyone at risk of a disease should do it especially those who are at risk of cancer. P11
	Apply	I think smoking and drinking alcohol plus online gaming and watching porn are addictive practices. These practices can get you into very harmful compulsion to use them and destroy your quality of life at the end. […] Additionally smoking causes lung and heart diseases and cancer. This is my view and knowledge on this although I am smoker myself; but I am on a plan to quit. P3
		I have taken the seasonal flu vaccine once because I have a chronic disease (…diabetes). P8

		[…] Regarding the flu vaccine, I used to get it for free from my employer. But I have never had it here. P10
		[…] I think it is very important. The earlier the better. My father had a heart condition so me and my siblings always do checkups for our heart functions. P12
		[…] vaccines are important and I regularly take the flu shot. P13
Health promotion	Access	"yes, healthy diet and keep quality sleeping." P1
		"[…] the collective responsibility is paramount. But it is not reachable all the time. Here in the Netherlands, the government put substantial effort on making the rich and the poor stay healthy. However, in the current circumstances in Yemen, you cannot hold the government accountable for these things. I mean there is ongoing war and insane amount of corruption, you can only spread awareness so people can stay healthy to the best of their ability." P1
		I think the individual should exercise and eat healthy food. P2
		our municipality has special support for people with low income to exercise. I think this a good way to make every able to be healthy. P2
		[…] it is a combination of things. As we discussed earlier. I really think that diet is very important. But I do not support extreme diets like stopping eating sugar at all. Just keep a bit of everything in it and do not over eat carbohydrate. It is also equally important to be fit and do not practice unhealthy behaviors such as smoking and drinking. I also do not think sporting as we see these days is a healthy behavior. I have been into a gym and I used to take protein supplements but it is actually not for health; I just realized that I am doing it for the sake of looking good. This is not the healthy point of exercising. Now I am just doing exercises that keeps me fit like running and swimming. I do not care about muscle building anymore. P3
		[…] I believe it is very important especially for the mental health of the workforce. I have been talking with a South Korean guy and he told me that people normally go over 60 hours a week. This is a bit too much and will eventually affect the health of the workers negatively. Luckily here in the Netherlands, they really look after the employees. P3
		[…] I fully agree with this. For example, in the Netherlands, they have very good food safety system but in Yemen we do not. This makes politics very important to facilitate healthy choices for the whole community. P3
		[…] this is true and important. You should keep healthy diet and avoid fast food and canned/packaged food. It is also important to keep sometime between the eating sessions and sleep well. […] our typical Yemeni diet is not also healthy. We eat so much rice and meat. That is why visceral overweight is common among some Yemenis. Now, I eat a bit of everything and I keep eating legumes, fruits and vegetables as well. P4
		, in Yemen, people usually eat sugar, rice and grain products because they are the most affordable. However, if the governments subsidizes growing fruits and vegetables, people will consider them into their regular diet. Personally, we were selling seasonal vegetables without profit when I was farming in Yemen with my family. P4
		[…] you should distract your attention from being in your mental problem state; for example, through exercising. However, you have to fix the main concern behind this mental state. […] You can also go to a psychologist to help you with this. P5
		yes, everyone should be. For example, eat health food such as fruits and vegetables, avoid too much sugars/fats/salt and be physically active. Also, you should pay attention to things that cause you mental troubles. P5
		keep the house well-ventilated, clean, exposed to sunlight and moderately warm. The bugs and rodents carry diseases but they are usually at home if it is not clean. P5
		just be very patient and face the struggles. […] I do not think going to the psychologist will help me. I have too many problems and I just feel ok confronting them with patience […e.g. it was really difficult and dangerous to get to Europe. I had to take the smuggling routes and I still remember that I had to burn my own jacket to get warm]. […] However, if you suffer from something that you cannot control at all and you cannot recover the normal state at all, then you can go to the psychologist. […] The stigma from the mental doctor (psychiatrist) in Yemen because people go there when they are totally out of normal mental state. So basically, only the insane people go there and that is why it is the doctor of "crazy people" and no one wants to be called crazy. P6
		of course, everyone should be responsible for his health. No one will take care of you properly if you do not know yourself properly. […] Diets are for people who have education on nutrition. Yemenis are usually not into diets. It is nearly impossible to see myself quitting my Yemeni diet; no matter what they say that we eat a lot and/or our diet has a lot of fats. […] I cannot imagine eating boiled dishes and brown bread all the day but I usually eat fruits and vegetables. P6
		[…] I think keeping balanced life is important. The lifestyle here is totally different from Yemen. I mean that people in Yemen may suffer from poverty and financial hardships which is a lot of stress. However, the kind of stress here is different. In Yemen we do not know that much about burnout but here it certainly exists. The way of studying and working here is really stressful. It is continuous, sometimes competitive and consumes too much time and thinking. This also may explain why some people commit suicides in the Netherlands. So I would say keep a balance in your life and do not over commit to studying or working. For example, you should have time for exercising, family and hobbies. […] Regarding the psychologist, I am against the common stigma towards psychologists/psychiatrists and people who go to them. However, I do not support going to the psychologist unless you feel the problem cannot solved by you. I know some people - who went to psychologists – suffered extra burden after the consultation. It is because the psychologist asked many different questions about different issues which provoked other traumatic experiences. Some of these people even resorted into drinking after going to the psychologist. I would say talk to your parents first and people who are close to you. P7
		[…] Everyone needs to be so by exercising, eating healthy and being mentally relaxed. […] Eating healthy means that you should go on a balanced diet: eating meat, fruits and vegetables. But you should be aware of over consuming meat, sugar and salt because they can cause hypertension and diabetes. It is funny that eating a lot of meat is a symbol of status in Yemen, though it causes diseases like arthritis. P7
		, it is not only on the shoulders of the individual. For instance, the Dutch government has ongoing policy on salt reduction on food products every year. Such polices show that the healthy choices are affected by the whole community and the government should facilitate that especially for needy ones. P7
		[…] things like stress can damage your health. I have diabetes and when I am stressed, the sugar levels in my blood go out of balance. […] It was hard for me to see that it is really difficult to work as I used to when I was in Yemen. This puts me under a lot of stress because you want to work here but the government offers you things that you do not enjoy doing. […] Things like having good spiritual relationship with God, exercising and staying close to your family and friends are good ways to prevent yourself from bad consequences of stress and depression. P8
		Everyone should go healthy before getting the problem. […] The healthy diet is key in this part. For example, if you eat everything without consideration you might end up like me having diabetes. […] I would usually go organic diet and products that are not processed. P8
		I can think of food safety and regulations as a very important tool that is outside the citizens' control but at the same time it is important to ensure healthy food for everyone. P8
		[…] it depends on the person but personally I keep socializing with my close people and keep myself busy with something I enjoy instead of over thinking. I would also spend time reading and watching documentaries.[…] Regarding the psychologist, if the person feels problem is serious, then he has to contact a psychologist. P9
		. […] You should follow healthy lifestyle and exercise. I believe everyone should find a hobby in sport and keep doing it even if he is old. In my opinion, exercising protects the body mentally and physically. P9
		[…] I think neighborhoods should have green areas and areas for exercising. P9
		[…] if the problem feels like a disease, the person should consult a psychologist. But generally, exercise relieves your mind by keeping you busy and helping you sleep. P10
		As I said, you should eat healthy, exercise, sleep well and avoid and manage stressful situations. […] You should eat fruits and vegetables and other foods especially the ones that contain protein. You should avoid eating a lot of carbohydrates and foods that causes overweight because otherwise you put yourself a higher risk of getting diabetes, hypertension and bad cholesterol. Additionally, you should eat less when you are old compared to when you are young because your physical activity decreases over time. P10
		Look here, I see that all the Dutch people can afford fruits and vegetables in their diet. It is probably the government helps them in terms of minimum wage and other income protections. […] The unemployed people in the Netherlands are offered paid sport memberships by the municipalities so the opportunity is available for everyone to be healthy. P10
		[…] well, it depends on the person. Personally, I feel that keeping spiritual practices, exercising and socializing with the people especially those who are close to you is very important to stand against mental issues and loneliness. P11
		[…] of course, the person should stay healthy in terms of eating and exercising. Also it is important to check your calorie and vitamin intake. You know that people who come from our region need to take care of their vitamin D intake here in the Northern hemisphere. P11
		the individual is responsible to a certain degree but the government is the primary actor in the healthy choices of the individual. For example, when there is no control over the safety of the food products, it would be really difficult for the individual to eat healthy food. Another example is what is going in Yemen, most of the people do not have income protections and cannot make decent wages. Therefore, they cannot afford balanced diet and buying fruits and vegetables regularly. P11
		[…] before my family moved to the Netherlands, I lived stressful days here. But I felt that when I am physically active especially riding my bike, I feel relieved. […] You also need to find a hobby to keep yourself busy such as painting or music. Additionally, the person should talk to the therapist or the psychologists if the problem is overwhelming. Going to the mental therapist is stigmatized in our culture. Many Yemenis will not go to the psychologist because of this even if they are depressed. All of this should be considered alongside healthy diet, exercising and keep close contact with your family and friends. […] I noticed that there is a lack of mental health awareness in the asylum centers. I saw many people depressed because of the ennui and long waiting. I think it would be useful if there are mental health awareness tailored to the people who come from cultures that stigmatize mental healthcare. P12
		You should be careful about your lifestyle choices so you do not need anyone to take care of you. […] For example, you should consume less sugar because it is almost everywhere in the diet; just be conscious. Also, we consume fruits and vegetables regularly because they are rich with the essential vitamins and nutrients. But this does not mean that you stop eating at restaurants; we eat pizza sometimes. P12
		When I talk to my brothers in Yemen about staying healthy. There are no places for exercising, the water is not safe and no proper regulations on food safety. Compared to the Yemen, I feel relatively comfortable in the Netherlands when I buy my son healthy snacks from the supermarket, we have green areas and nice exercising facilities. Here you see it as a community health not only a personal matter. P12
		[…] I believe that exercising is very important and it is proven to help you cope with stress and mental issues. You can also go to a mental therapist, talk with social supporters and your close people. These are useful ways to prevent bad mental health problems especially for refugees due to trauma, separation from family and the ongoing crises in the home country. P13
		People should treat their body well by exercising, eating healthy, quitting smoking and avoiding things that cause stress. […] You should reduce your sugar and fat consumption, and keep balanced diet and regular intake of fruits and vegetables. P13
		[…] indeed, you do not really have a free choice when it comes to health. Not everyone has the resources and the facilities to exercise and eat healthy so the governments should help with this. P13
	Understand	"[…] eat three to four times a day with sufficient time lag. Also you should eat balanced diet with vegetables and fruit, expose yourself to the sun to get vitamin D and avoid fatty foods. Sleeping is also so important because if you stay all night, you probably would not sleep well in the day and your diet will be very messy which may lead you to eat fast foods and enter a cycle of unhealthy stuff. Additionally, eating natural and fresh food is healthier than the packaged food. Also I really dislike hydrogenated fats (trans fats)." P1
		"[…] first of all you should address anything that cause you stress and/or make you depressed. I also believe reading and learning new stuff like languages makes your brain busy. Plus exercising is important and great way to release the pressure and spend time."P1
		"[…] I think you should keep your home free from bugs, dust and rodents. Also keep it well-ventilated and let the sun light enter the home." P1
		I actually do not know how to read these label. I just exercise, join gym, eat vegetables and be mentally relaxed. But I think exercise burns the bad foods that I eat. P2
		you try to eat healthy and avoid sugars. This includes eating organic food and avoiding packaged food. It is also important to read and be aware about your mental problem. Playing sports and killing time is effective too. […] Another issue is that we have grown in a culture where psychological and psychiatric treatment are stigmatized, but I think they are very helpful when it is too much to handle. P3
		[…] to be honest with you, I am not aware of these labels. I see them and I rarely see what is in these. I read the ingredients but not how much of certain nutrients is there and what is the healthy amount for me. I just try to make it organic: avoiding packaged/canned food and always eating fruits and vegetables. For example, I buy dry beans over the canned one. P3
		[…] unfortunately no. But I think keeping healthy diet is the most important thing alongside exercising. […] So I do not eat until I get too full and I am subscribing to a local gym. P4
		[…] keeping the house clean and well-ventilated is important to make you healthy physically and mentally. I also do not like being in a shared properties because you cannot 100% control others behavior with regard to cleaning and the overall housing conditions. P4
		[…] the most important thing is to keep the house clean and well-ventilated. I cannot imagine leaving the house unclean for two days, it will really make me feel stressed out. P6
		[…] I do not really read everything. However, everyone can know how much food they need per day from the family doctor or over the internet. Fortunately, I do not suffer unhealthy diet problems and I keep a balanced diet with my family by eating meat in few occasions per week and keeping vegetarian diet most of the week. P7
		[…] I really do not read them. But I always go for the organic and fresh products. P13
		[…] the employees should not feel under too much pressure and stress. This may increase the output for short period of time but it will damage the productivity in the long run. It is also interesting to see some employers offer sport facilities inside the work place. More importantly, the atmosphere should be healthy in the workplace; for example, it should be free from snitching and bullying. P13
	Process	. I sometimes come home with emails and stuff from wok to complete at home. This is annoying and stressful. I think it is because of some people at the management in my workplace: they make you feel pressured to do a lot of things so you can keep your spot with them. I would feel insecure if I do not do these extra tasks. This is mentally unhealthy. P2
		It is nice that the Netherlands have green areas. Lanes for normal people and people with special needs. Public parks and facilities for children and adults. However, I do not think this is the only thing to create healthy environment. I lived in two different places here. But it is socially very different from where I grew up in Yemen. Neighbors here are distant from each other and barely do something together. I do not think they trust each other that much. I think when neighbors are more closer to each other, they will live in harmony and healthier atmosphere especially mentally. It has been so strange for me to treat or be treated by some neighbors so formally. P2
		of course everyone wants to live in a clean and safe neighborhoods. Good sanitary system and accessible and open playgrounds for everyone are examples of that. But we have a problem regarding this. We do not have enough sunlight entering our building, which is important to get vitamin D. It is because we are surrounded with tall college campus that makes the sunlight goes only between 1-4 hours a day over the four seasons. P3
		first of all, there should be less stress because that will leave the workers vulnerable to health problems. Secondly, the atmosphere between the workers should be free from unhealthy behaviors such as bullying and snitching. P5
		[…] the work place should be relaxing for the employees. For example, I have visited companies where there are places to take naps and the employer provide fruit and vegetable snacks. […] However, it is essential to keep the workload manageable for the employees and the work environment is free from stress and bullying. P7
		[…] I fortunately work at a place where they give us exercise allowance. This is never in Yemen. The work-life balance is really considered here so we stay away from pressure and stress as much as possible. There are also standing tables at the work place. I really feel good about my work. P9
		, I believe that the government should control such things because it will produce returns for the whole society. Healthier employees means less health resources consumed and more productivity. P10
		You know that people who come from our region need to take care of their vitamin D intake here in the Northern hemisphere. P11
		[…] this there is an important link between health and productivity: if you are healthy physically and mentally, you will be more productive. In the Netherlands, there is a great deal of attention towards the working hours, overnights and protecting the worker's health in general. Unfortunately, the employees are not really protected in Yemen especially those who work at the private sector. P11
	Apply	"[…] no I do not. I do not trust them. I just try to avoid these packaged foods and go organic. You do not know about these expiry dates; many chemical stuff inside. It is just simple for me: if I want beans, I would buy them in their organic form and I will process them. The same for the other things." P1
		"[…] yes, but not right now. I am busy with work these lately. I go swimming every now and then." P1
		No, but I jog, walk and exercise myself. P5
		Food labels: […] I never looked at them. P6
		This is my weak point. But it is always in my mind and I usually use the bike. I had a plan few weeks ago to get a membership from a nearby fitness club and swimming lessons but everything ruined because of the Coronavirus crisis. P7
		Food labels:[…] this is difficulty for Yemeni people. We just eat till we feel full. But it is always good to see the doctor or dietician to help you keep a healthy diet. P8
		[…] I tried to once but I quit. It was far from home. But I usually walk and exercise myself. P8
		[…] I have a membership in a local gym. I go regularly there.P9
		honestly I do not read the food labels, though I have lately started reading them for my children health. […] I also noticed that the diet in my region in Yemen is mainly based on grains and that is maybe why the average height is low and the children are slightly stunted over there. P9
		[…] I do not really read them. But my wife is on a diet and keeps reading them. However, I avoid eating canned/packaged food. P10
		[…] I do not really look at them. Fortunately, my BMI is within the normal range and this is the reason I do not look at the labels. However, I believe that everyone should avoid over eating carbohydrates and fats. P11
		[…] I joined multiple gyms in the past but I cannot do it constantly because of the weather and work. Additionally, I regularly play soccer and take my son to swimming and martial arts lessons. P11
		[…] I yes I read them. I usually read them to avoid high sugar and calorie products and foods that contain a lot of preservatives. P12
		[…] Yes, I have joined a gym. We do aerobic and strength exercises. P12
		[…] yes I have a membership with a gym in the neighborhood. But I cannot go the gym now because of Coronavirus so I have an outdoor jogging routine. P13
